# E4BP4 facilitates glucocorticoid-evoked apoptosis of human leukemic CEM cells via upregulation of Bim

**DOI:** 10.1186/1750-2187-6-13

**Published:** 2011-10-05

**Authors:** Jessica A Beach, Laura J Nary, Yasuko Hirakawa, Eli Holland, Rebeka Hovanessian, Rheem D Medh

**Affiliations:** 1 Department of Biology, California State University Northridge, Northridge, CA 91330-8303, USA

## Abstract

**Background:**

Synthetic GCs serve as therapeutic agents for some lymphoid leukemias because of their ability to induce transcriptional changes via the GC receptor (GR) and trigger apoptosis. Upregulation of the BH3-only member of Bcl-2 family proteins, Bim, has been shown to be essential for GC-evoked apoptosis of leukemic lymphoblasts. Using human T cell leukemic sister clones CEM-C7-14 and CEM-C1-15, we have previously shown that the bZIP transcriptional repressor, E4BP4, is preferentially upregulated by GCs in CEM-C7-14 cells that are susceptible to GC-evoked apoptosis, but not in refractory CEM-C1-15 cells. E4BP4 is an evolutionarily conserved member of the PAR family of bZIP transcription factors related to the *C. elegans *death specification gene *ces2.*

**Results:**

Mouse E4BP4 was ectopically expressed in CEM-C1-15 cells, resulting in sensitization to GC-evoked apoptosis in correlation with restoration of E4BP4 and Bim upregulation. shRNA mediated modest knockdown of E4BP4 in CEM-C7-14 cells resulted in concomitant reduction in Bim expression, although GC-evoked fold-induction and sensitivity to apoptosis was similar to parental cells.

**Conclusion:**

Data presented here suggest that GC-mediated upregulation of E4BP4 facilitates Bim upregulation and apoptosis of CEM cells. Since the Bim promoter does not contain any consensus GRE or EBPRE sequences, induction of Bim may be a secondary response.

## Background

Glucocorticoids (GCs) are known to evoke human lymphoid cell apoptosis [1-3] primarily by binding to and modulating the transcriptional activity of the GC receptor (GR) [4]. GCs possess immunosuppressive and anti-inflammatory properties and serve as effective therapeutic agents for different forms of leukemia [5], asthma, rheumatoid arthritis, and irritable bowel syndrome [6]. In order to exploit the full therapeutic potential of GCs, GC/GR-mediated gene regulation and its impact on various cellular processes needs to be better understood. To this end, we and others have studied GR-dependent gene regulation by microarray-based transcriptional profiling [7-9]. A subset of genes were identified as those being upregulated selectively in human leukemic CEM cells susceptible to, but not in cells refractory to, GC-evoked apoptosis [7]. In this report, one of those genes, E4BP4, was evaluated for its role GC-evoked apoptosis.

E4BP4 (adenovirus E4 binding protein 4), also called NFIL3 (nuclear factor, interleukin 3 regulated) is classified as a mammalian basic leucine zipper (bZIP) transcription factor and is closely related to the PAR (proline and acid rich) sub-family of bZIP transcription factors, although it lacks a PAR domain [10]. Vertebrate PAR family transcription factors include hepatic leukemia factor (HLF), D-box binding protein (DBP), and thyrotroph embryonic factor (TEF) [11]. While other PAR family members activate transcription, E4BP4 represses transcription by binding to the same DNA sequence (E4BP4 response element; EBPRE), whose consensus sequence is (G/A)T(G/T)A(C/T) GTAA (C/T) [10]. The repressing activity of E4BP4 has been attributed to a small 65 amino acid C-terminal repression domain that is rich in charged residues [10,12]. There are instances where it activates transcription of target genes as well [11].

Orthologs of PAR family proteins include C. *elegans *Ces-2 [13], D. *melanogaster *Vrille [14], and X. *laevis *Gene8 and Gene9 [15], which are known to have crucial functions in apoptosis, morphogenesis, and tail resorption. E4BP4 has been implicated in diverse functions, including regulation of circadian rhythms [16], osteoblast function [17], motoneuron survival [18], protection of B cells from apoptosis induced by IL-3 deprivation [19], IgE class switching [20], and NK cell development [21]. Interestingly, E4BP4 has been shown to exhibit both pro-apoptotic and pro-survival functions in a cell- and stimulus-specific fashion. For example, IL-3-mediated survival of pro-B cells is facilitated by the upregulation of E4BP4 [19], while the antitumor properties of cantharidin have been attributed to its ability to upregulate E4BP4 and inhibit the antiapoptotic properties of HLF [22]. Owing to its repressive activity, E4BP4 has been suggested to function as an antagonist to other PAR family transcription factors, which compete to bind to the same DNA sequences [23].

E4BP4 has been shown to bind the TBP-binding repressor protein Dr1 and facilitate its ability to repress both basal and activated transcription [24]. There is evidence that PAR proteins follow a pathway analogous to their ortholog in *C. elegans*, Ces-2, which is known to down regulate the survival gene Ces-1, which subsequently permits the upregulation of the proapoptotic gene Egl-1 [13,25,26]. PAR family proteins, including E4BP4, have been shown to modulate the activity of Egl-1 orthologs, the pro-apoptotic BH-3 only members of the Bcl-2 family, either directly or via Ces-1 orthologs Slug and Snail [27,28].

BH3-only proteins of the Bcl-2 family, Bim and Puma, are required for the initiation of apoptosis by multiple stimuli, including γ-radiation, oxidative stress and GCs [29-31]. Bim is required for negative selection of T cells and B cells, and for termination of T cell immune response [32]. Puma has been identified as a p53-inducible gene and is thought to be critical for DNA-damage induced apoptosis [33]. In CEM cells, induction of Bim is essential for GC-evoked apoptosis, and was one of the genes identified through microarray-based expression profiling, along with E4BP4, as being selectively upregulated in response to GCs in the GC-sensitive sister subclones of CEM cells [7]. In this report, we present evidence that E4BP4 plays a crucial role in GC-evoked apoptosis of CEM cells by enabling induction of Bim.

## Results

Previous work has demonstrated the sensitivity and resistance of CEM-C7-14 and CEM-C1-15 cells, respectively, to GC-evoked cell death via apoptosis [7]. The resistance in CEM-C-15 cells is thought to occur because of a blunted GR-dependent transcriptional response, hence several studies have focused on identifying key transcriptional changes that are unique to GC-sensitive CEM cell clones [7-9]. E4BP4 was identified as one of the key genes upregulated in correlation with GC-evoked apoptosis [7] only in GC-sensitive CEM lines through microarray-based expression profiling. To investigate the role of E4BP4 in GC-evoked apoptosis, CEM cells were manipulated either to overexpress (CEM-C1-15) or knockdown (CEM-C7-14) E4BP4.

### Creation of a clone of CEM-C1-15 cells expressing mouse E4BP4 transcript

**Figure 1:**
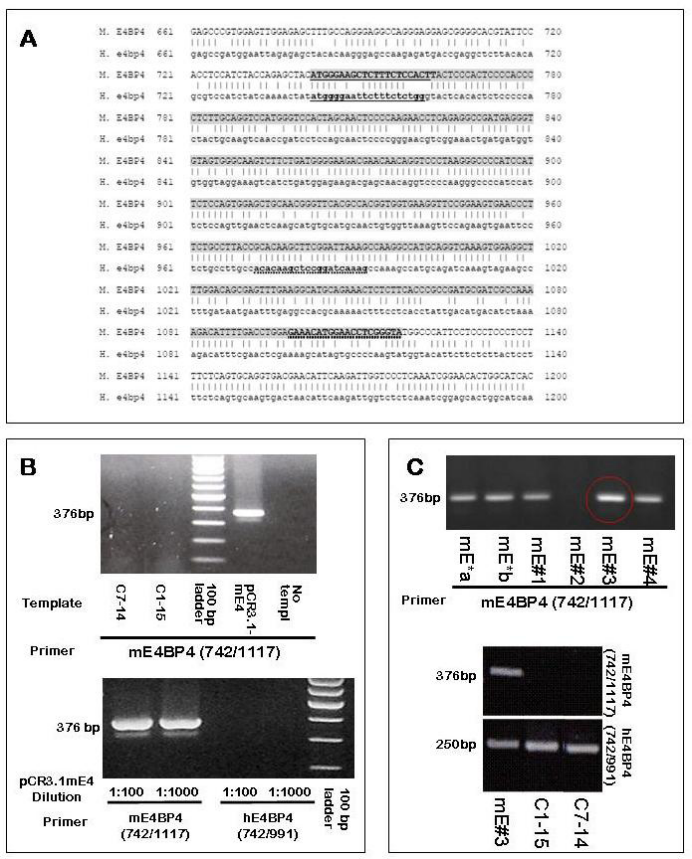
**Primer Specificity for human and mouse E4BP4 and generation of M.E4BP4 expressing CEM C1-15mE#3 cells**: **Panel A**: Sequence alignment of partial mouse and human E4BP4 coding sequences within exon 2 (numbering is based on +1 for the first nucleotide of the coding region). Upper rows indicate mouse E4BP4 sequence in upper case and lower rows show human E4BP4 sequence in lower case. Forward and reverse primers are underlined with solid and dotted lines respectively (mE4BP4 = 742/1117 and hE4BP4 = 742/991). **Panel B**: The top gel shows specificity of mE4BP4 primers for M. E4BP4 template. PCR using DNA extracted from human CEM clones failed to amplify any product. The positive control template of plasmid pCR3.1-mE4 amplified a 376bp fragment. For the lower gel, 1:100 and 1:1000 dilutions of the plasmid DNA pCR 3.1-mE4BP4 were subjected to qRT-PCR analysis using primers mE4BP4 (742/1117) and hE4BP4 (742/991). Product amplification was observed only with mE4BP4 (742/1117). **Panel C**: CEM-C1-15 cells electroporated with 13.5 μg of linearized pCR3.1-mE4 plasmid expressing M. E4BP4 were selected in the presence of 400 μg/ml Geneticin. Seven micrograms of total RNA extracted from two mass culture populations (mE*a and mE*b) and four clones (mE#1-mE#4) obtained by limiting dilution was subjected to reverse transcription followed by end-point PCR analysis for the expression of M. E4BP4 (top gel). The clone labeled mE#3 (CEM C1-15 mE#3) (circled) was used for further analysis. Lower gel shows PCR products obtained with both mE4BP4 (742/1117) and hE4BP4 (742/991) primers using reverse transcription products from clone mE#3, parental CEM-C1-15 and CEM C7-14 cells as the template.

The mouse and human E4BP4 genes are highly homologous, with 79% nucleotide sequence identity in the coding region. The two proteins are 462 amino acids long and have an 84% sequence identity and 92% sequence homology, based on a BLASTP pairwise sequence alignment algorithm. To determine whether a lack of E4BP4 upregulation was contributing to GC resistance in CEM-C1-15 cells, a construct expressing M. E4BP4 (pCR3.1-mE4) was transfected into CEM-C1-15 cells, allowing for selective amplification of either the human or mouse transcript using species-specific primers. Although both proteins are almost identical, we cannot rule out the possibility that the mouse protein may have unique characteristics compared to the human version. For both species, the entire coding sequence of E4BP4 is within a single exon. As shown in Figure 1A, regions of sequence variation within the partial coding sequence shown were utilized to design PCR primers for species-specific amplification, and mouse (376bp) and human (250bp) specific amplicons were identified based on their sizes. As shown in Figure 1B, the mE4BP4 primers failed to amplify any product from CEM-C7-14 or CEM-C1-15 cells (top panel), whereas the hE4BP4 primers failed to amplify any product when the plasmid pCR3.1-mE4 was used as a template (bottom panel). As shown in Figure 1C, upper panel, two batches of mass cultures of M. E4BP4 transfected CEM-C1-15 cells (mE*a and mE*b) as well as four different clones generated by limiting dilution were tested for presence of transcript corresponding to mouse E4BP4 sequences. Except for clone 2 (mE#2), the mass cultures and all clones had amplicons corresponding to M. E4BP4 transcript. Clone #3 (mE#3) was chosen for further analysis, and as shown in Figure 1C, lower panel, it exhibited both mouse and human specific E4BP4 transcript by reverse transcription PCR analysis.

### Transfection of M. E4BP4 in CEM-C1-15 cells restores sensitivity to Dex-evoked apoptosis

**Figure 2:**
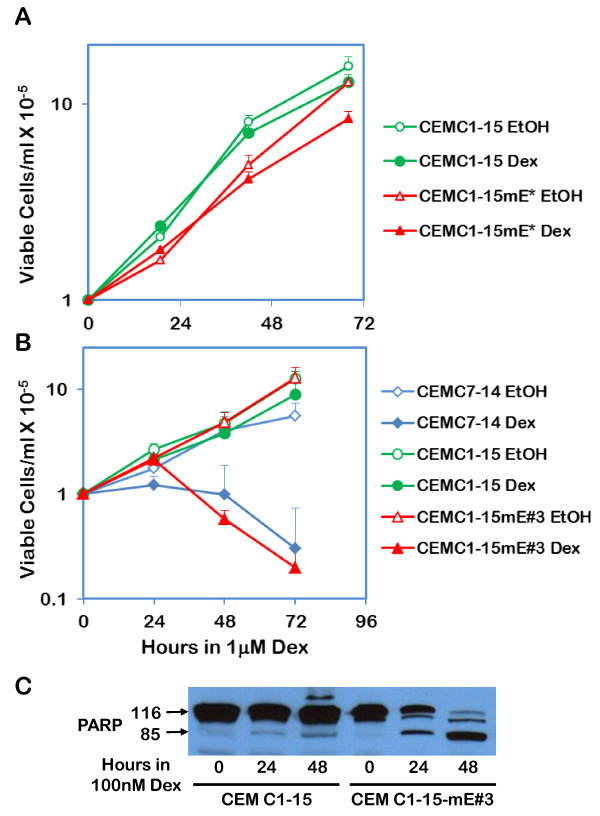
**Dex induces apoptosis in CEM C1-15 cells transfected with M. E4BP4**: **Panel A**:CEM C1-15 and a mass culture CEM C1-15 cells (CEM C1-15mE*) transfected with the mouse E4BP4 expressing construct pCR3.1-mE4 were seeded at a density of 1 × 10^5^cells/ml and treated for 72 h with either 0.1% ethanol (EtOH) or 1 μM Dex. Aliquots were taken at 24 h intervals and viable cell number was determined by trypan blue dye exclusion assay. Data represent average ± S.D. of three independent experiments with two replicates each. **Panel B**:A clone of CEM C1-15mE* with confirmed expression of mE4BP4 (CEM C1-15mE#3), and parental CEM C1-15 and CEM C7-14 cells were analyzed for cell viability in the presence of 0.1% ethanol or 1 μM Dex as in Panel A. Data represent averages ± S.D. of three separate experiments with two replicates each. **Panel C**: CEM C1-15 or CEM C1-15mE#3 cells treated for 48 h with 100 nM Dex and cell lysates were prepared from aliquots harvested every 24 h. Protein content of lysates was determined by the Bradford assay and 30 μg total protein from each sample was evaluated for PARP cleavage by Western blotting using a C-terminal-specific anti-PARP antibody (SC-7150, Santa Cruz Biotechnology).

Parental CEM-C1-15 cells, mass cultures of cells transfected with M.E4BP4 (CEM-C1-15mE*), and clone # 3 (CEM-C1-15-mE#3) were tested for their sensitivity to 1 μM Dex by the trypan blue dye exclusion assay. As expected, CEM-C1-15 cells were resistant to Dex induced cytotoxicity, while CEM-C1-15mE* cells showed a slight reduction (18%) in viable cell number after 68h in 1 μM Dex (Figure 2A). The cloned line, CEM-C1-15-mE#3, exhibited Dex-evoked sensitivity similar to the sensitive CEM-C7-14 cells, with viable cell numbers after 72h in 1 μM Dex being less than 5% of the corresponding ethanol treated cells (Figure 2B). To test whether Dex activated apoptosis in CEM-C1-15-mE#3 cells, lyastes of cells treated with ethanol or 100nM Dex were analyzed for PARP cleavage by Western blotting using an anti-PARP antibody that recognizes both the intact protein (116kDa) and the cleaved C-terminal portion (85kDa). After 48h in 100nM Dex, parental CEM-C1-15 cells showed no significant accumulation of the cleaved 85kDa band, while the CEM-C1-15-mE#3 cells showed a majority of PARP as the 85kDa band, suggesting caspase-dependent cleavage, and hence activation of apoptosis. All three lines were subjected to cytogenetic analysis. Previous data suggests that a key difference between the two sister clones is that CEM-C7-14 cells bear a t(1;2)(p13;q21) translocation, while the CEM-C1-15 cells do not [7]. Karyotype analysis (data not shown) confirmed that CEM-C1-15mE#3 cells and parental CEM-C1-15 cells had normal chromosomes 1 and 2, while CEM-C7-14 cells had the translocation. These data ruled out the possibility the CEM-C1-15-mE#3 cells were a population of CEM-C7-14 cells inadvertently mixed with the CEM-C1-15 cells that were transfected with M.E4BP4, and reiterating that transfection of M.E4BP4 may have contributed to the responsiveness of CEM-C1-15mE#3 cells to Dex-induced cell death.

### Accumulation of cells in sub-G1 phase in response to Dex

**Figure 3:**
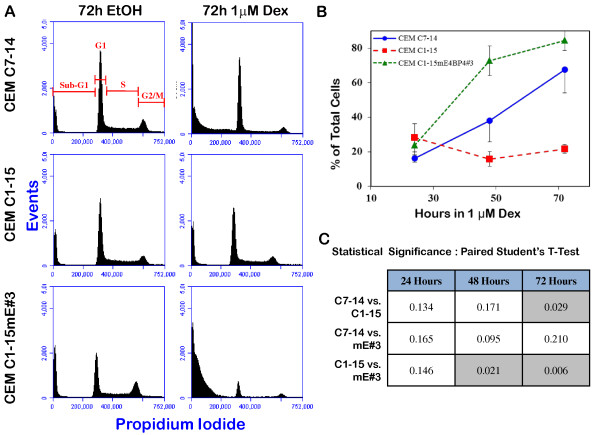
**Flow cytometric cell cycle analysis demonstrates sub-G1 accumulation of CEM C1-15mE#3 cells treated with Dex**: CEM C7-14, CEM C1-15 and CEM C1-15mE#3 cells were treated with 0.1% ethanol (EtOH) or 1 μM Dex for 24, 48 and 72 h, stained in propidium iodide, and analyzed for cell cycle distribution flow cytometrically using an Accuri C6 flow cytometer and CFlow^® ^software. **Panel A**: Representative histograms of cell cycle distribution of ethanol and Dex treated cells after 72 h treatment. For each analysis 50,000 singlet cells were gated into sub-G1, G1, S and G2/M as indicated for the top left histogram. **Panel B**: Time course of accumulation of cells with sub-G1 DNA content as a percentage of total cells analyzed. Average ± S.D. from three independent experiments. **Panel C**: Data from three independent experiments were subjected to a paired Student's T-test (two sample, equal variance). Gray shaded boxes represent significant differences with p-values < 0.05.

DNA content was measured by flow cytometric analysis of propidium iodide stained cells at 24, 48 and 72 h after treatment with either ethanol or 1 μM Dex (Figure 3). Accumulation of cells with sub-G1 DNA content was apparent after 48 h in Dex. In CEM-C7-14 and CEM-C1-15mE#3 cells, exposure to 1 μM Dex for 48 h resulted in approximately 38% and 73% of the cells with DNA content in the sub-G_1 _range, respectively, while cells treated with ethanol had only 15% and 20% of the cells with sub-G_1 _DNA content. Exposure to 1 μM Dex for 72 h shifted 68% and 85% of CEM-C7-14 and CEM-C1-15mE#3 cells to sub-G1 phase, respectively. In GC-resistant CEM-C1-15 cells only moderate changes in sub-G_1 _cell accumulation were observed, with 14% and 22% of cells in sub G1 phase after 72 h in ethanol and 1 μM Dex, respectively. It is interesting to note that the CEM-C1-15mE#3 cells appear more sensitive to Dex than the CEM-C7-14 cells, which correlates to the results observed in a Dex titration viability assay (data not shown).

### GC-dependent upregulation of Bim is restored in CEM-C1-15mE#3 cells

**Figure 4:**
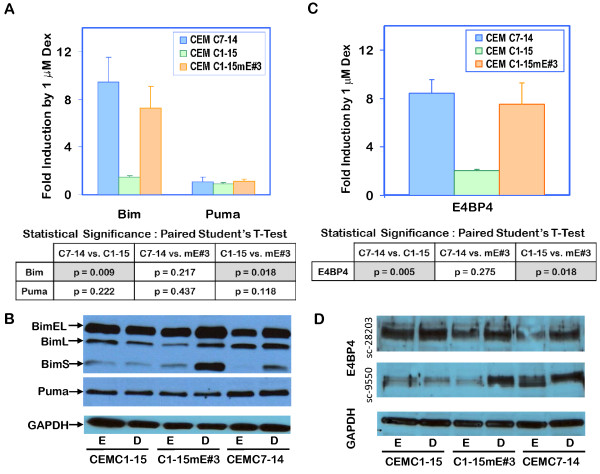
**Dex-mediated upregulation of Bim and E4BP4 is restored in CEM C1-15mE#3 cells**: **Panels A and C**: CEM C7-14, CEM C1-15 and CEM C1-15mE#3 cells were treated with 0.1% ethanol or 1 μM Dex for 24 h and total RNA was extracted in TriZol. Seven microgram RNA was subjected to reverse transcription reaction and real-time qPCR using primers specific for Bim or Puma (Panel A) or E4BP4 (Panel C) as listed in Table 1. Fold change in expression of each transcript by 1 μM Dex was calculated by the Pfaffl method using β-actin as a reference. Data represent averages ± S.D. from three independent experiments, which were analyzed for statistical significance using a paired student t-test, as shown in the table below each bar chart. Shaded boxes represent significant differences with p-values ≤ 0.05. **Panels B and D**: CEM C7-14, CEM C1-15 and CEM C1-15mE#3 cells were treated with 0.1% ethanol (E) or 100 nM Dex (D) for 24 h and total protein was extracted in lysis buffer. Protein content of lysates was measured by the Bradford assay, and 30 μg protein from each sample was evaluated by Western blotting for Bim and Puma expression (Panel B) or for E4BP4 expression using two different antibodies from Santa Cruz Biotechnology (Panel D). An antibody for GAPDH was used as a reference.

Upregulation of Bim [34] and Puma [31] have been implicated in Dex-evoked apoptosis of CEM-C7-14 and other lymphoid cells. In GC-sensitive CEM-C7-14 cell, Bim upregulation has been shown to contribute to apoptosis, while in GC-resistant CEM-C1-15 cells, GCs do not upregulate either Bim or Puma. To determine whether restoration of GC-induced apoptosis in CEM-C1-15mE#3 cells correlated with restoration of Bim upregulation, all three CEM lines were treated with either ethanol or 1 μM Dex for 24 h and analyzed for Bim and Puma transcript levels by reverse transcription followed by RT-qPCR, as detailed in the methods section. As reported previously, CEM-C7-14 cells responded to Dex by almost a 9.5-fold induction of Bim expression, while in CEM-C1-15 cells, no significant induction was observed. In CEM-C1-15mE#3 cells, Bim transcript levels were induced by over 7-fold in the presence of 1 μM Dex (Figure 4A). Puma expression was not significantly affected by Dex in any of the cell lines tested. Bim protein levels were evaluated by Western blotting using an antibody that recognizes all three isoforms of Bim: BimEL, BimL and BimS. As seen in Figure 4B, both CEM-C7-14 and CEM-C1-15mE#3 cells showed Dex-dependent induction of BimEL and BimS protein levels, while CEM-C1-15 cells were non-responsive. Interestingly, CEM-C1-15mE#3 cells showed a markedly higher fold-induction based on band intensity, which correlates with the greater sensitivity of these cells to Dex-evoked accumulation of cells in Sub-G1 phase (Figure 3A).

### GC-mediated E4BP4 upregulation is restored in CEM-C1-15mE#3 cells

Since E4BP4 seems to be a key transcriptional regulator that may alter expression of several genes involved in GC-mediated apoptosis, changes in E4BP4 transcript levels were also measured in all three cell lines in response to 1 μM Dex. As expected, CEM-C7-14 cells showed a 8.4-fold induction of E4BP4 transcript levels, while CEM-C1-15 cells showed a minimal 2-fold increase (Figure 4C). Unexpectedly, in CEM-C1-15mE#3 cells, Dex induced a 7.5-fold increase in E4BP4 expression (Figure 4C). Since the primers used were specific for H. E4BP4 and do not detect the transfected M. E4BP4 (Figure 1B), these data demonstrate that transfection of M. E4BP4 somehow facilitates GR-dependent transcriptional regulation of endogenous E4BP4. These data emphasize the importance of E4BP4 regulation in GC-evoked apoptosis. In Western blotting experiments using two different E4BP4-specific antibodies that recognize both the mouse and human homologs, it is clear that E4BP4 protein levels are not affected by Dex in CEM-C1-15 cells, but are induced in CEM-C7-14 and CEM-C1-15mE#3 cells (Figure 4D). Since basal E4BP4 protein levels are high in CEM-C1-15 cells compared to CEM-C7-14 cells (top panel, Figure 4D), transfection of M. E4BP4 did not significantly alter total protein abundance in EtOH treated samples, however, introduction of M.E4BP4 restored Dex-dependent induction of E4BP4 protein (2-3 fold in different experiments). Our data suggest that in CEM-C1-15 cells, E4BP4 protein levels are deregulated, and that regulation is restored upon M. E4BP4 transfection.

### Dex-mediated effects in CEM C1-15mE#3 cells are GR-dependent

**Figure 5:**
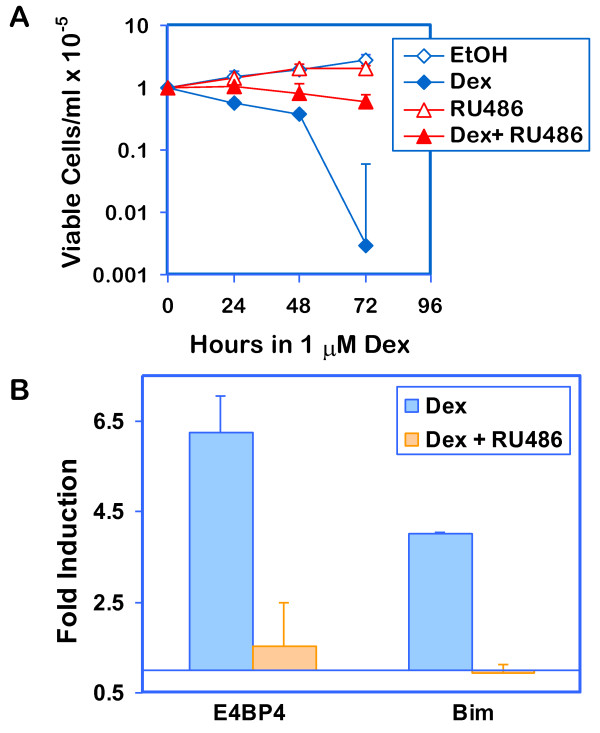
**RU38486 blocks Dex-mediated effects in CEM C1-15mE#3 cells **: **Panel A: **CEM C1-15mE#3 cells were seeded at a density of 1 × 10^5^cells/ml and treated for 72 h with either 0.1% ethanol (EtOH), 1 μM Dex, 1 μM RU486, or both Dex and RU486. Trypan blue excluding viable cells were counted at 24h intervals. Data represent averages ± SD from triplicate sets of treatments from two experiments. **Panel B**: CEM C1-15mE#3 cells were treated as in Panel A for 24h and RNA was extracted with TriZol. RNA was subjected to reverse transcription reaction and real-time qPCR using primers specific for Bim or Puma as described for Figure 4. Fold change in expression was calculated by the Pfaffl method using β-actin as a reference. Data represent averages ± SD from two independent experiments run in duplicates.

To confirm that Dex-dependent cell death and E4BP4 and Bim upregulation are dependent on GR, the effects of the GR antagonist RU 38486 (RU486) on cell viability and induction of transcription were monitored. RU486 blunted Dex-evoked loss of cell viability (Figure 5A), and prevented Dex-mediated upregulation of H.E4BP4 and Bim transcripts (Figure 5B).

### shRNA-mediated E4BP4 knockdown shows correlation between E4BP4 and Bim expression

**Figure 6:**
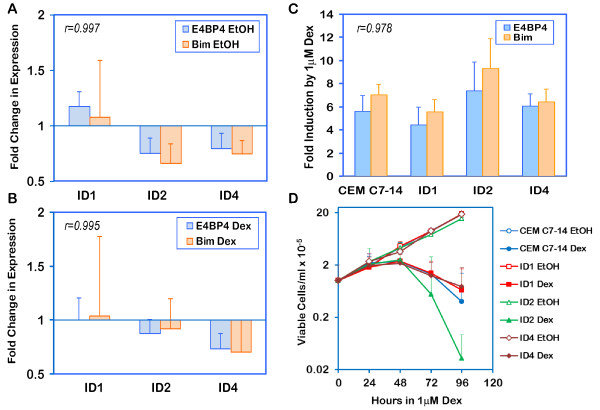
**shRNA based variable knockdown of E4BP4 correlates with corresponding repression of Bim expression**: CEM C7-14 cells were transfected with three different E4BP4 shRNA plasmidsID1, ID2 and ID4 with a puromycin selection marker. Transfected cells were selected in the presence of 1 μg/ml puromycin, and surviving cells were analyzed for extent of E4BP4 knockdown and Bim expression in comparison to untransfected cells. Cells were treated with 0.1% ethanol or 1 μM Dex for 24h and total RNA was extracted in TriZol. Seven microgram RNA was subjected to reverse transcription reaction and real-time qPCR using primers specific for E4BP4 or Bim as listed in Table 1. Fold change in expression in knockdown cells (ID1, ID2 or ID4), compared to CEM C7-14 cells, for ethanol treated (Panel A) and Dex treated (Panel B) cells was calculated by the Pfaffl formula: (E_target_)^ΔCTtarget^/(E_ref_)^ΔCTref ^where ΔCTtarget = (CT_CEM C7-14_-CT_ID_) for E4BP4 or Bim and ΔCTref = (CT_CEM C7-14_-CT_ID_) for β-actin. Induction of E4BP4 or Bim expression in response to 1 μM Dex was calculated for CEM C7-14, ID1, ID2, and ID4, by the Pfaffl method, whereΔCTtarget = (CT_ethanol_-CT_Dex_) for E4BP4 or Bim and ΔCTref = (CT_ethanol_-CT_Dex_) for β-actin (Panel C). Efficiency for each reaction was calculated using the software LinRegPCR. Data represent averages ± S.D. from three independent experiments. Correlation between E4BP4 and Bim expression was determined by calculating the correlation coefficients (r) which are indicated on each panel. Panel D: CEM C7-14 and mass cultures of cells transfected with ID1, ID2 and ID4 shRNA plasmids against E4BP4, and selected with puromycin were seeded at a density of 1 × 10^5^cells/ml and treated for 96 h with either 0.1% ethanol (EtOH) or 1 μM Dex. Aliquots were taken at 24 h intervals and viable cell number was determined by trypan blue dye exclusion assay. Data represent average ± S.D. of three independent experiments with two replicates each.

It is noteworthy that GC-resistant CEM-C1-15 cells express abundant basal E4BP4 transcript and protein, but fail to respond to GCs by upregulating either E4BP4 or Bim. To determine the effect of E4BP4 knockdown on Bim expression and sensitivity to GCs, CEM-C7-14 cells stably transfected with three different E4BP4-specific shRNA plasmids, ID1, ID2 and ID4, were created. As shown in Figure 6A, two of these cell populations, ID2 and ID4 showed about 25% knockdown in the basal (ethanol treated) state, when compared to the parental CEM-C7-14 cells, while ID1 showed marginally higher E4BP4 expression than CEM-C7-14 cells. This pattern closely correlated with the extent of basal Bim expression, with both ID2 and ID4 showing 33 to 25% reduction in basal expression compared to CEM-C7-14 cells, and ID1 showing a marginal increase in Bim expression compared to parental cells. Similarly, when E4BP4 transcript levels were compared in Dex-treated cells, ID2 and ID4 showed 12% and 27% reduction, respectively, when compared to CEM-C7-14 cells, while ID1 showed no change (Figure 6B). In correlation, Bim transcript levels were 8% and 30% reduced in ID2 and ID4 cells, when compared to CEM-C7-14 cells, in response to Dex, and ID1 did not show any change. Thus, although the extent of knockdown achieved was poor, the data demonstrated a clear correlation between E4BP4 and Bim expression, with correlation coefficients greater than 0.995 in both ethanol and Dex treated groups. Based on E4BP4 expression levels in parental CEM-C7-14 and CEM-C1-15 cells, it is apparent that rather than absolute abundance of E4BP4 and Bim transcripts, the change in expression in response to Dex determines sensitivity. Thus, each of the shRNA transfected cell populations, and CEM-C7-14 cells were also evaluated for their ability to upregulate E4BP4 and Bim expression in response to 1 μM Dex. As shown in Figure 6C, all cell populations, including the knockdowns, showed a 4.5 to 7.5-fold upregulation of H. E4BP4 expression in response to Dex treatment, with a parallel 5.6 to 9.4-fold Dex-induced upregulation of Bim expression. Remarkably, ID2 cells showed a greater increase in E4BP4 and Bim expression compared to CEM-C7-14 cells. The E4BP4 knockdown cells were evaluated for their viability in the presence of 1 μM Dex (Figure 6D), and all cells were susceptible to Dex-evoked cell death, comparable to CEM-C7-14 cells. Interestingly, ID2 cells, which showed the maximum fold induction of E4BP4 and Bim expression, also showed the greatest sensitivity to Dex-evoked cell death, confirming the correlation between E4BP4/Bim inducibility and susceptibility to cell death.

## Discussion

GCs are essential components of various therapeutic regimens because of their immunosuppressive and lymphoid cell apoptosis-inducing properties [35]. GC-evoked apoptosis of lymphoid cells is triggered via GR-dependent transcriptional regulation of key pro- and anti-apoptotic genes [36-38]. Previous studies have established E4BP4 as one of approximately forty genes that are upregulated following Dex exposure in the GC-sensitive CEM-C7-14 subclone [7]. E4BP4 is a 'delayed-early-response' gene which is upregulated by several stimuli, including IL-3 [39], IL-4 [40], calcium [41,42], cAMP [43], and GCs [44], and is a known transcriptional repressor with homology to the C. elegans pro-apoptotic ces-2 gene and the mammalian PAR family genes [11,18].

The PAR family proteins have distinct functions in cell survival and apoptosis, and E4BP4 has been reported to be a survival factor for pro-B cells [19], cardiomyocytes [45], and motoneuron cells [18]. In contrast, E4BP4 expression is upregulated by the tumor suppressor PTEN, and is suppressed in ovarian cancers [46], suggesting that it is a pro-apoptotic factor. Indeed, our data suggests that E4BP4 is a proapoptotic factor in T-lymphoid leukemic cells, because expression of ectopic M.E4BP4 sensitizes CEM-C1-15 cells to GC-evoked apoptosis. It has been previously established that CEM-C7-14 and CEM-C1-15 cells possess approximately equal numbers of GR sites per cell, however, CEM-C1-15 cells possess a lower binding affinity between GCs and the GR than CEM-C7-14 cells, and a blunted GR-dependent transcriptional response [47]. E4BP4 may stabilize the GC-GR transcription complex, and ectopic E4BP4 expression may restore GR-dependent transcription and therefore reestablish GC sensitivity in CEM-C1-15mE#3 cells. Human E4BP4 expression was significantly induced in CEM-C1-15mE#3 cells following Dex exposure, with levels comparable to the induction observed in CEM-C7-14 cells, suggesting that E4BP4 may be involved in an autoregulatory loop, with ectopic expression of the mouse gene restoring regulation of the endogenous gene. When CEM-C7-14 cells were transfected with E4BP4 shRNA, upto 25% knockdown of basal and Dex-induced E4BP4 expression, over parental CEM-C7-14 cells was achieved. However, the fold-induction in the presence of Dex (comparing basal and Dex-induced expression within each knockdown group) was comparable to parental CEM-C7-14 cells, explaining why shRNA-mediated knockdown did not cause CEM-C7-14 cells to become refractory to Dex-evoked apoptosis. Two lines of evidence indicate that CEM-C1-15mE#3 cells are truly derived from CEM-C1-15 cells: karyotype analysis and PCR-array-based transcriptional profiling both suggest these cells to be distinct from CEM-C7-14 cells and related to CEM-C1-15 cells (data not shown).

Upregulation of Bim is required for Dex-induced cell death in CCRF-CEM cells. Knockdown of Bim expression by shRNA gene silencing strongly reduced cell death in response to Dex treatment [48]. In data presented here, Dex-mediated Bim induction was restored in CEM-C1-15mE#3 cells in conjunction with their sensitization to Dex-evoked apoptosis upon expression of ectopic M. E4BP4. These data suggest that Bim expression may be regulated by E4BP4, or that ectopic E4BP4 enables GR-dependent transcriptional responses, which include Bim upregulation. In CEM-C7-14 cells transfected with E4BP4 shRNA, reduction of basal or Dex-induced E4BP4 expression (when compared to similarly treated parental CEM-C7-14 cells) by about 25%, correlated with a parallel reduction in basal and Dex-induced Bim expression by about 30%, strongly suggesting that Bim is a downstream target of E4BP4-mediated transcriptional regulation. Within each knockdown group, the fold-induction in Bim expression after Dex treatment (comparing basal to Dex-induced expression) was similar to parental CEM-C7-14 cells, hence there was minimal effect on cell viability. These data suggest that the relative change in expression, rather than the absolute amount of expression, of E4BP4 and Bim determine sensitivity to GC-evoked apoptosis. Promoter analysis of Bim did not indicate the presence of an EBPRE or GRE, which suggests E4BP4 or GR do not directly regulate Bim expression. However, E4BP4 could regulate Bim expression through a yet to be determined intermediate, or through the formation of a ternary complex with another transcription factor on the Bim promoter. Recent studies have proposed that PAR bZIP proteins have a role in the transcriptional control of BH3-only proapoptotic genes. Benito et al. showed that promoter for *bcl-gs *(a BH3-only gene) is responsive to TEF activation and is silenced by E4BP4 in human tumor cells [28,49].

E4BP4 has been shown to induce differentiation in monocyte-macrophages [50], to drive natural killer cell lineage development [51] and to regulate IgE class-switching [20], all important immunological responses. E4BP4 has also been shown to antagonize the function of other PAR family proteins, namely, HLF, TEF and DBP, owing to the presence of a repressor domain in E4BP4 competing for the same or similar DNA binding sequences [23,52]. Studies have implicated HLF and TEF as antiapoptotic factors [53,54], and leukemic stem cells have been shown to consistently overexpress HLF [55,56]. Moreover, microarray profiling has identified HLF as a "stemness" gene in hematopoietic stem cells [57,58]. E4BP4, by virtue of its ability to antagonize HLF, has been suggested to regulate the anti-apoptotic properties of HLF [23,59]. In fact, cantharidins, a class of potential anti-tumor agents, are thought to induce apoptosis by E4BP4-mediated inhibition of the antiapoptotic activity of HLF [55]. It is possible that HLF and other PAR family proteins play an important role in CEM cell apoptosis, and that the ratio of E4BP4 and other PAR proteins regulates the apoptotic state of these cells.

## Conclusions

Studies presented here strongly suggest an important role for E4BP4 in leukemic T-cell apoptosis through activation of the Bim pathway. E4BP4 may modulate GR-dependent transcription, either by binding directly to GR, or to one of its coregulatory factors. Alternatively, E4BP4 may activate a repression pathway, such that it may repress a negative regulator of Bim, facilitating enhanced Bim transcription.

## Methods

### Reagents

Dexamethasone (Dex) was purchased from EMD Biosciences (Madison, WI). The GR antagonist RU38486 was a gift from Dr. E. Brad Thompson (UTMB, Galveston, TX). Reagents for reverse transcription (RT) and Real-time qPCR, including M-MLV reverse transcriptase, oligo(dT)_15 _primer, RNasin^®^Ribonuclease inhibitor, dNTP mix, and Taq DNA polymerase were purchased from Promega Life Sciences (Madison, WI). SYBR^®^JumpStart™*Taq*ReadyMix was from Sigma-Aldrich (St. Louis, MO). Other reagent grade chemicals were purchased from Fisher Scientific (Pittsburgh, PA) or Sigma-Aldrich.

### Cell culture and treatments

The CCRF-CEM [60] derived human T-ALL cell lines CEM C7-14 and CEM C1-15 are sensitive and resistant to GCs, respectively, and are generous gifts from Dr. E. B. Thompson, University of Texas Medical Branch, Galveston. The cells were cultured in RPMI 1640 (with L-glutamine) from Cellgro(Manassas, VA, Catalog #50-020-PB) supplemented with 5% heat-inactivated fetal bovine serum (FBS) from Atlanta Biologicals (Lawrenceville, GA, Cat #S11150). Cells were maintained in log phase at 37°C in a 5% CO_2 _incubator. Cell treatments were for 24 to 96 h in RPMI supplemented with 5% FBS containing either 100 nM or 1 μM Dex (diluted from a 1000x stock in ethanol) or 0.1% ethanol as vehicle alone.

### Estimation of cell viability

Cells were plated at a density of 1 × 10^5 ^cells/ml and treated for 96 h with 0.1% ethanol or 1 μM Dex diluted from a 1000x stock prepared in ethanol. Aliquots were removed at 24 h intervals for cell counts. Viable cells were counted by the trypan blue exclusion method using a Hemocytometer.

### Primer design

Mouse (GenBank accession # NM_017373) and human (accession # NM_005384) E4BP4 cDNA sequences were aligned to identify regions of maximum mismatch (Figure 1A). Species-specific forward primers from the same region (starting at nucleotide 742) and staggered reverse primers were designed to allow for amplicons of 376bp and 250bp for mouse- and human-specific PCR products, respectively, as indicated in Figure 1A. Primer specificity was confirmed as indicated in Figure 1B. The three isoforms of human Bim: BimS, BimL, and BimEL, differ in amino acid length, but all possess the characteristic BH3 domain within exon five. The Bim primers were designed across exons five and six to detect and amplify all three transcripts concurrently. These and additional primers used for quantitation of Puma and β-actin are listed in Table 1.

**Table 1:** Primers used for PCR

Transcript (Primer Name)	Forward Primer	Reverse Primer	Product Size
H. E4BP4 (HUMANE4BP4-742 & 991)	5'ATGGGGAATTCTTTCTCTGG3'	5'CTTTGATCCGGAGCTTGTGT3'	250 bp

M. E4BP4 (Mouse E4BP4-742 &1117)	5'ATGGGAAGCTCTTTCTCCACT3'	5'TACCCGAGGTTCCATGTTTC3'	376 bp

Bim (BIM 5/6 SENSE & ANTI)	5'CAGATATGCGCCCAGAGATA3'	5'ACCAGGCGGACAATGTAAC3'	163 bp

Puma (PUMA SENSE & ANTI)	5'AAGAGCAAATGAGCCAAACG3'	5'GCAGAGCACAGGATTCACAG3'	181 bp

β-actin (B-ACTIN-130 SENSE & ANTI)	5'AGTCCTCTCCCAAGTCCACA3'	5'CACGAAGGCTCATCATTCAA3'	130 bp

### Transfection of M. E4BP4

The M. E4BP4 expressing construct pCR 3.1-mE4BP4 (generous gift from Dr. Sotirios Tetradis, UCLA) was linearized with Sca I and resuspended 10 mM TrisHCl, pH 8.0. Logarithmic phase CEM C1-15 cells were resuspended in 400 μl of serum free RPMI to a final density of 5 × 10^6 ^cells/ml, mixed with 13.5 μg of linearized pCR 3.1-mE4BP4, andelectroporated at 1050 μF and 260 volts in the BioRad Gene Pulser II. Following electroporation, the cells were allowed to recover for 48 h in 2 ml of pre-warmed RPMI media, supplemented with 5% FBS. Transfected cells were selected in 400 μg/ml Geneticin, and a preliminary analysis of surviving cells revealed presence of mouse E4BP4-specific transcript (Figure 1C). This mass culture was cloned by limiting dilution to establish multiple clonal lines of CEM C1-15 cells expressing M. E4BP4. The clonal line CEM C1-15mE#3 was used for further analysis.

### Reverse transcription and RT-qPCR (real-time-quantitative PCR) analysis

Cells were treated at a density of 4 × 10^5 ^cells/ml for 24 h with either ethanol or Dex, and RNA was extracted from approximately 1 × 10^7 ^cells using the TRIzol reagent (Invitrogen Life Technologies, La Jolla, CA). For first-strand DNA synthesis, 7 μg of total RNA was reverse transcribed for 3 h at 42°C in the presence of 0.5 μg of oligo(dT)_15_, 1 μl (~200 U) of M-MLV reverse transcriptase, 0.5 mM dNTP mix, and 100 U of RNase inhibitor. For RT-qPCR, 1 μl of the reverse transcription product was mixed with SYBR^® ^Green JumpStart™ Taq Ready Mix(Sigma-Aldrich, Cat #4438) and the appropriate primers (Table 1) in a final volume of 25 μl, and run on a Cepheid SmartCycler (Sunnyvale, CA). To quantitate the relative expression levels, the cycle threshold (CT) values for each sample were used to calculate fold inductions using the Pfaffl method [61] formula: (E)^ΔCTtarget(control-sample)^/(E)^ΔCTreference(control-sample)^, where β-actin was the reference gene. E represents primer efficiency, which was calculated for each sample reaction using the freeware program LinRegPCR. Statistical analysis was done on Excel using a paired Student's t-test, where p ≤ 0.05 was considered to be statistically significant.

### Analysis of Cell Cycle Distribution by Flow Cytometry

Cells were seeded at a density of 1.0 × 10^5 ^cells/mL in 5-25 ml of media, and treated with ethanol or 1.0 μM Dex for 24, 48, or 72 hours prior to fixing. Cells were washed twice in PBS, resuspended in 0.5 ml of low salt stain (30 mg/ml PEG 6000, 25 μg/mlpropidium iodide, 0.01% Triton-X-100, and 0.01% RNase A in 4 mM sodium citrate), and incubated at 37°C for 20 minutes. Following incubation, 0.5 ml of high salt stain (30 mg/ml PEG 6000, 25 μg/ml propidium iodide, 0.01% Triton-X-100, and 0.01% RNase A in 400 mM sodium chloride) was added to the samples and mixed by gentle vortexing. After a brief storage at 4°C, samples were analyzed for propidium iodide staining on an Accuri C6 Flow Cytometer^®^(Accuri Cytometers Inc., Ann Arbor, MI). Data were processed using the Accuri CFlow^® ^software, gating to include only single cells. Extent of propidium iodide staining of the gated population was displayed in a histogram and the following regions were defined: sub-G_1_, G_1_, S, and G_2_/M. Percentages of cells in each region was calculated from three independent experiments. Standard deviation and probability by Student T-tests were calculated in Excel.

### Western blotting

Cells plated at a density of 4 × 10^5 ^cells/ml were treated for 24 h or 48 h with the ethanol or Dex, and approx. 8 × 10^6 ^cells were harvested, washed and lysed in buffer containing 50 mM Tris-HCl, pH 7.4, 150 mM NaCl, 1% NP-40, plus a protease inhibitor cocktail. The amount of protein in each sample was estimated using the Bradford assay, and 30 μg of each sample was boiled in SDS-PAGE sample buffer (final composition: 120 mM Tris, 4% SDS, 20% glycerol, 5% 2-mercaptoethanol, 0.05% bromophenol blue, pH 6.8) Samples were resolved on a 10% or 12.5% polyacrylamide-SDS gel, and electoblotted on to PVDF membranes. Membranes were blocked in 10% non-fat dry milk and incubated sequentially with appropriate primary and secondary antibodies. Polyclonal antibodies (cat#s sc-9550 and sc-28203) that recognize both mouse and human E4BP4, and anti-PARP antibody (cat# sc-7150) that recognizes both the full-length and truncated versions of the protein, were from Santa Cruz Biotechnology (Santa Cruz, CA). Polyclonal anti-Bim antibody (cat# 559685) which recognizes BimS, BimL and BimEL was from BD-Pharmingen (San Diego, CA), polyclonal anti-Puma antibody (cat# AP1317a) was from Abgent (San Diego, CA), and polyclonal anti-GAPDH antibody (cat# 2118) was from Cell Signaling Technology (Beverly, MA). Secondary horseradish peroxidase (HRP) conjugated anti-rabbit IgG and anti-goat IgG were from Santa Cruz Biotechnology (Santa Cruz, CA). Membranes were developed using an Enhanced Chemiluminescence (ECL) kit from Pierce Biotechnology (Rockford, IL).

### shRNA based E4BP4 knockdown

Five micrograms each of four pre-designed shRNA plasmids (ID1 to ID4) from SA Biosciences, each containing a different short hairpin RNA (shRNA) sequence targeting human E4BP4, and containing a puromycin resistance gene, were electroporated separately into2 × 10^6 ^CEM-C7-14 cells (in 250 μl) at 1050 μF and 260 volts, using the IngenioTM Electroporation reagent (Mirus Bio, Madison, WI). Puromycin resistant populations from three transfectants, ID1, ID2 and ID4 could be selected in media containing 1 μg/ml puromycin, while ID3 transfected cells failed to survive. Efficiency of E4BP4 knockdown in ethanol and 1 μM Dex treated cells compared to similarly treated parental CEM C7-14 cells, and extent of Dex-dependent upregulation of E4BP4 and Bim was determined by RT-qPCR analysis. Data were processed from three independent experiments using the Pfaffl method, as detailed in the legend for Figure 5. To determine the relationship between E4BP4 expression and Bim expression, correlation coefficients were calculated for each set of data. Effect of Dex on cell viability in shRNA transfected cells was determined in three independent experiments by trypan blue dye exclusion assay.

## Competing interests

The authors declare that they have no competing interests.

## Authors' contributions

JAB, LJN and RDM were involved in experimental design and data analysis. JAB, LJN and RH performed the experiments and helped with preparation of figures. YH and EH participated in critical analyses of data and provided expert technical advice. RDM prepared the manuscript and served as the principal investigator. All authors have read and approved the final manuscript.
